# Identification of intraoperative management strategies that have a differential effect on patients with reduced left ventricular ejection fraction: a retrospective cohort study

**DOI:** 10.1186/s12871-022-01817-z

**Published:** 2022-09-10

**Authors:** Michael D. Maile, Michael R. Mathis, Elizabeth S. Jewell, Graciela B. Mentz, Milo C. Engoren

**Affiliations:** 1 Department of Anesthesiology, University of Michigan, 4172 Cardiovascular Center, 1500 E. Medical Center Drive, Ann Arbor, MI USA; 2 The Max Harry Weil Institute for Critical Care Research and Innovation, Ann Arbor, MI USA

**Keywords:** Anesthesiology, Left ventricular dysfunction, Postoperative complications, Remifentanil

## Abstract

**Background:**

There are few data to guide the intraoperative management of patients with reduced left ventricular ejection fraction (LVEF). This study aimed to describe how patients with reduced LVEF are managed differently and to identify and treatments had a different risk profile in this population.

**Methods:**

We performed a retrospective cohort study of adult patients who underwent general anesthesia for non-cardiac surgery. The effect of anesthesia medications and fluid balance was compared between those with and without a reduced preoperative LVEF. The primary outcome was a composite of acute kidney injury, myocardial injury, pulmonary complications, and 30-day mortality. Multivariable logistic regression was used to adjust for confounders. Treatments that affected patients with reduced LVEF differently were defined as those associated with the primary outcome that also had a significant interaction with LVEF.

**Results:**

A total of 9420 patients were included. Patients with reduced LVEF tended to have a less positive fluid balance. Etomidate, calcium, and phenylephrine were use more frequently, while propofol and remifentanil were used less frequently. Remifentanil affected patients with reduced LVEF differently than those without (interaction term OR 2.71, 95% CI 1.30–5.68, *p* = 0.008). While the use of remifentanil was associated with fewer complications in patients with normal systolic function (OR 0.54, 95% CI 0.42–0.68, *p* < 0.001), it was associated with an increase in complications in patients with reduced LVEF (OR = 3.13, 95% CI 3.06–5.98, *p* = 0.026).

**Conclusions:**

Patients with a reduced preoperative LVEF are treated differently than those with a normal LVEF when undergoing non-cardiac surgery. An association was found between the use of remifentanil and an increase in postoperative adverse events that was unique to this population. Future research is needed to determine if this relationship is secondary to the medication itself or reflects a difference in how remifentanil is used in patients with reduced LVEF.

**Supplementary Information:**

The online version contains supplementary material available at 10.1186/s12871-022-01817-z.

## Background

Heart failure is a devastating syndrome that often has a prognosis worse than many types of cancer [1]. Management focuses on slowing progression, management of symptoms, and the appropriate use of mechanical support. Given its association with postoperative adverse events, surgery can be avoided when possible [2, 3]. However, with the consistent incidence and increasing survival, the number of patients with heart failure presenting for surgery continues to increase making it vital that we continue to improve our ability to care for these patients perioperatively [4].

Reduced left ventricular ejection fraction (LVEF) accounts for approximately half of heart failure diagnoses. The presence of reduced LVEF confirms at least stage B heart failure (structural heart disease without signs or symptoms) according to the latest ACC/AHA guidelines [5]. This has been shown to be an important and independent risk factor for patients undergoing non-cardiac surgery, even if symptoms of heart failure are absent [6, 7]. Since there are no high-grade recommendations for how to manage patients with reduced LVEF undergoing non-cardiac surgery, anesthesiologists must extrapolate data from non-surgical studies to care for these patients.

Given differences between acute physiologic stress and long-term disease progression, extrapolating data from studies of the long-term management of heart failure to the perioperative period may lead to unintended consequences. Therefore, studies are needed to define the optimal perioperative management of these patients. The objective of this study was to identify any intraoperative medications or fluid administration strategies that affect patients with reduced ejection fraction differently than other patients. We accomplished this by finding management strategies that had a significant interaction with reduced LVEF after adjusting for other patient and surgical characteristics. We hypothesized that the use of certain medications or fluid management strategies are associated with a different level of risk for patients with reduced LVEF compared to those with a normal LVEF.

## Methods

### Study population

This retrospective cohort study was approved by the Institutional Review Board, which waived the requirement for written informed consent. The study was presented at a departmental research forum prior to accessing data and the RECORD statement of the STROBE guidelines were followed for reporting the results. All patients had a general anesthetic for a non-cardiac surgical procedure at a single tertiary-care medical center between January 2008 and April 2019 and had an echocardiographic assessment of their LVEF within 1 year prior to surgery.

To create a homogenous study population, patients were excluded if they received blood products during the surgery. Non-operative anesthesia cases that were done for medical (endoscopy or cardiology procedures) or interventional radiology cases were excluded given their low baseline risk and common use of monitored anesthesia care instead of general anesthesia. Neuraxial anesthetics were excluded given their low use. Patients with hyperdynamic LVEFs or undergoing emergent surgery were also excluded.

### Exposure

Medications were included if they were administered to greater than 5% and less than 95% of cases to have enough individuals in each group to detect an effect. Opioids, such as morphine, fentanyl, and hydromorphone, were converted to oral morphine equivalents and analyzed as a single medication [8]. Remifentanil was analyzed separately given its rapid metabolism. Medications were analyzed as a binary variable while the intraoperative fluid balance was analyzed as a continuous variable calculated by subtracting the estimated blood loss and the urine output from the volume of crystalloid recorded in the medical record.

### Covariates

**Fig. 1:**
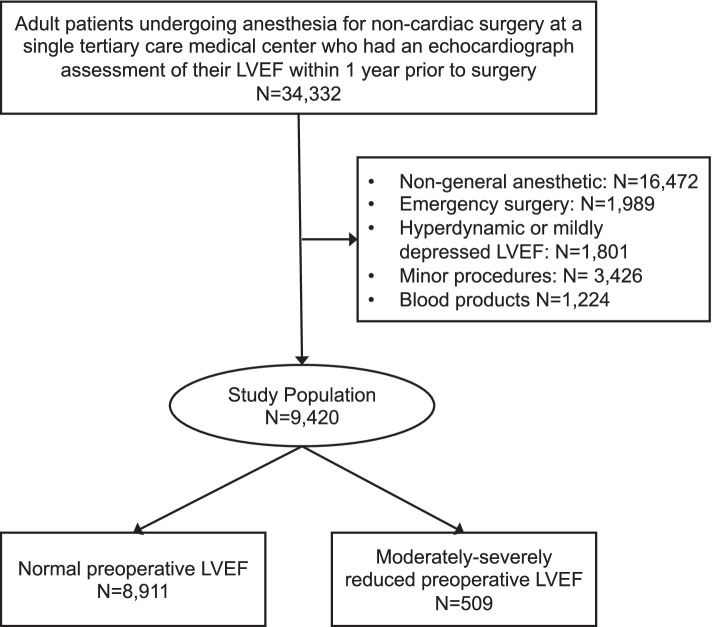
Derivation of study population

Baseline patient characteristics and surgical attributes were obtained from the medical record. These consisted of the subject’s age, sex, Elixhauser-based comorbidity summary measure [9], surgical type, surgical duration, and postoperative hemoglobin [10]. Surgical duration was divided into short (≤180 minutes) and long (> 180 minutes). Surgery types were empirically collapsed into three surgery risk categories (Supplemental Fig. 1). Intraoperative intravenous fluids were administered at the discretion of the clinical team. No guidelines existed recommended a minimum amount of resuscitation during the time of this study. Echocardiographic data were obtained from the institutional echocardiography database. The results of these studies are in the electronic medical record and are routinely reviewed by the anesthesia providers before the procedure. In accordance with the latest recommendations of the American Society of Echocardiography [11], LVEF was considered normal if it was within the range of 54–74% for females and 52–72% for males. Mildly reduced ejection fraction was defined as 41–53% for females and 41–51% for males. Moderately and severely reduced ejection fraction were defined as 30–40 and < 30%, respectively, for both sexes. Patients with moderately or severely reduced LVEF were combined into one group and compared to patients with normal LVEF (Fig. 1).

### Outcomes

The primary outcome was a composite of pulmonary complications, acute kidney injury, myocardial injury, and 30-day mortality. Mortality was included in the composite outcome because it represents a competing risk with the other postoperative complications [12]. The occurrence of pulmonary complications was derived from International Classifications of Disease (ICD) codes for the following: pulmonary edema (ICD-9: 518.4, ICD-10: J81.0), respiratory arrest (ICD-9: 799.1, ICD-10: R09.2), acute respiratory failure (ICD-9: 518.81; ICD-10: J95.821, J95.822, J96.00–.02, J96.90–.92), respiratory insufficiency (ICD-9:518.5, 518.51, 518.52, 518.53; ICD-10: J95.1, J95.2, J95.3), other respiratory complications (ICD-9: 514, 977.0%, 506.0, 506.1, 506.2, 506.3, 507%; ICD-10: J81.0, R09.0%, J68.0, J68.1, J68.2, J68.3, J68.8, J69.%, J95.4). These definitions were predefined by the Michigan Perioperative Outcomes Group to be consistent with those used by the Agency for Healthcare Research and Quality [13]. Acute kidney injury (AKI) was identified by an increase in serum creatinine by ≥0.3 mg/dL within 48 hours of anesthesia end time or a ≥ 50% increase within seven postoperative calendar days. Myocardial injury was defined as a postoperative troponin level that was above the laboratory reference limit for the assay. Finally, the occurrence of mortality within 30 days of surgery was obtained from the medical record.

### Statistical analysis

Initial statistical exploratory data analysis techniques were used to assess the central tendency, dispersion, and frequency distribution of measures, outcomes, predictors, and confounders. Given the large sample size, all continuous variables were summarized as the mean ± standard deviation. Categorical variables were summarized using counts and percentages. Extreme values were identified and investigated to determine whether they represented erroneously recorded data that should be removed. Missing data patterns and rates were examined, and it was decided that complete case analysis was the most appropriate choice.

Differences between cohort characteristics were quantified using the standardized difference (SD) given the large sample size using the *stddiff* macro in SAS [14]. A SD greater than 0.2 was used to identify unbalanced distribution of confounders between patient groups. Given the substantial number of medications, principal component analysis was used as a dimension reduction technique that allowed for inspection of initial associations. Since the principal components did not simplify the interpretation of the intraoperative management of patients with and without reduced LVEF, each medication was analyzed individually. These were analyzed along with patient and surgical factors to find medications that had a different association in patients with reduced LVEF compared those with a normal LVEF.

Multivariable logistic regression was used to adjust for potential confounders. Variables included in multivariable logistic models were checked for multi-collinearity using the following criteria: if Pearson’s correlation index was greater than 0.7 and the variance inflation factor (VIF) was greater than 10 the variable was removed. Given the retrospective nature of the study, use of a matched sample was assessed by examination of distribution of confounders by LVEF level using standardized differences (SD) (Table 1). It was concluded that there was no need to use a matched sampling strategy. Variables with SD greater than 0.1 were included in the multivariable models. Least absolute shrinkage and selection operator (LASSO) was used for variable selection and regularization [15]. To minimize confounding by indication, only intraoperative management factors with a statistically significant interaction with reduced LVEF, were considered clinically significant. *p*-value < 0.05 and a 95% confidence interval (CI) that excludes 1 were used to denote statistical significance.

**Table 1:** Subject characteristics

	Complete Population (***N*** = 9420)	Normal LVEF (***N*** = 8911)	Reduced LVEF (***N*** = 509)	
**Patient and Surgical Characteristics**	**Mean (STD)**	**Mean (STD)**	**Mean (STD)**	**SD**
Age (years)	58.8 (15.3)	58.7 (15.3)	62.0 (15.3)	0.22
BMI (kg/m^2^)	29.8 (7.7)	29.9 (7.8)	28.4 (7.0)	0.21
Surgery Duration (min)	186.2 (106.0)	186.5 (106.2)	182.0 (103.4)	0.04
	**N (%)**	**N (%)**	**N (%)**	**SD**
Race				0.13
American Indian or Alaska Native	29 (0.3)	28 (0.3)	1 (0.2)	
Asian or Pacific Islander	204 (2.2)	196 (2.2)	8 (1.6)	
Bi- or Multi-Racial	5 (0.1)	5 (0.1)	0 (0.0)	
Black, not Hispanic	921 (9.8)	868 (9.7)	53 (10.4)	
Middle Eastern	14 (0.2)	13 (0.2)	1 (0.2)	
Unknown race	627 (6.7)	579 (6.5)	48 (9.4)	
White, not Hispanic	7620 (80.9)	7222 (81.1)	398 (78.2)	
Sex				
Female	4957 (52.6)	4783 (53.7)	174 (34.2)	0.40
Male	4463 (47.4)	4128 (46.3)	335 (65.8)	
WHO Obesity Classification				0.02
Underweight	186 (2.0)	172 (2.0)	14 (2.8)	
Normal Weight	2328 (25.1)	2189 (24.9)	139 (28.2)	
Overweight	2948 (31.7)	2765 (31.4)	183 (37.1)	
Class I Obesity	1933 (20.8)	1837 (20.9)	96 (19.5)	
Class II Obesity	1028 (11.1)	992 (11.3)	36 (7.3)	
Class III Obesity	864 (9.3)	839 (9.5)	25 (5.1)	
ASA Physical Status Classification				0.04
1	139 (1.5)	139 (1.6)	0 (0.0)	
2	2595 (27.6)	2575 (28.9)	20 (3.9)	
	**N (%)**	**N (%)**	**N (%)**	**SD**
3	5648 (60.0)	5378 (60.4)	270 (53.1)	
4	1034 (11.0)	815 (9.2)	219 (43.0)	
Admission Type				0.56
Admit	4026 (42.7)	3859 (43.3)	167 (32.8)	
Inpatient	2169 (23.0)	1931 (21.7)	238 (46.8)	
Outpatient	3225 (34.2)	3121 (35.0)	104 (20.4)	
Comorbidities				
CAD	1872 (19.9)	1601 (18.0)	271 (53.2)	0.81
AIDS or HIV	23 (0.2)	23 (0.3)	0 (0.0)	0.07
Alcohol Abuse	171 (1.8)	157 (1.8)	14 (2.8)	0.07
Anemia from Blood Loss	199 (2.1)	181 (2.0)	18 (3.5)	0.09
Arrhythmia	2010 (21.3)	1774 (19.9)	236 (46.4)	0.61
COPD	1859 (19.7)	1712 (19.2)	147 (28.9)	0.23
Coagulopathy	599 (6.4)	539 (6.1)	60 (11.8)	0.21
CHF	1134 (12.0)	791 (8.9)	343 (67.4)	1.62
Iron Deficiency Anemia	507 (5.4)	469 (5.3)	38 (7.5)	0.09
Depression	1447 (15.4)	1343 (15.1)	104 (20.4)	0.14
Diabetes	1878 (19.9)	1721 (20.5)	157 (32.6)	0.28
Fluid & Electrolyte Disorders	1712 (18.2)	1540 (17.3)	172 (33.8)	0.40
Hypertension	4706 (50.0)	4373 (52.2)	333 (69.2)	0.35
Hypothyroidism	1130 (12.0)	1051 (11.8)	79 (15.5)	0.11
Liver Disease	627 (6.7)	588 (6.6)	39 (7.7)	0.04
Lymphoma	323 (3.4)	302 (3.4)	21 (4.1)	0.04
Obesity	2091 (22.2)	2009 (22.6)	82 (16.1)	0.17
Other Neurologic Disorders	734 (7.8)	670 (7.5)	64 (12.6)	0.17
Paralysis	248 (2.6)	233 (2.6)	15 (3.0)	0.02
PUD	76 (0.8)	72 (0.8)	4 (0.8)	0.00
PVD	1275 (13.5)	1113 (12.5)	162 (31.8)	0.50
Psychoses	115 (1.2)	105 (1.2)	10 (2.0)	0.06
Renal Failure	1473 (15.6)	1313 (14.7)	160 (31.4)	0.42
	**N (%)**	**N (%)**	**N (%)**	**SD**
Solid Tumor Without Metastasis	2331 (24.8)	2228 (25.0)	103 (20.2)	0.12
Surgery Risk				0.03
Low	5717 (60.7)	5471 (61.4)	246 (48.3)	
Moderate	3245 (34.5)	3025 (34.0)	220 (43.2)	
High	458 (4.9)	415 (4.7)	43 (8.5)	
Duration				0.03
Short	6006 (63.8)	5675 (63.7)	331 (65.0)	
Long	3414 (36.2)	3236 (36.3)	178 (35.0)	
Surgery Type				< 0.01
Acute Care & Trauma	458 (4.9)	415 (4.7)	43 (8.5)	
General Surgery	2293 (24.3)	2204 (24.7)	89 (17.5)	
Neurosurgery	714 (7.6)	687 (7.7)	27 (5.3)	
Orthopedics	1261 (13.4)	1211 (13.6)	50 (9.8)	
Otolaryngology	729 (7.7)	675 (7.6)	54 (10.6)	
Plastic Surgery	431 (4.6)	414 (4.7)	17 (3.3)	
Thoracic	991 (10.5)	950 (10.7)	41 (8.1)	
Urology & Gynecology	1732 (18.4)	1642 (18.4)	90 (17.7)	
Vascular	811 (8.6)	713 (8.0)	98 (19.3)	

*AIDS* Acquired Immunodeficiency Syndrome, *ASA* American Society of Anesthesiologists, *BMI* Body Mass Index, *CAD* Coronary Artery Disease, *CHF* Congestive Heart Failure, *COPD* Chronic Obstructive Pulmonary Disease, *HIV* Human Immunodeficiency Virus, *LVEF* Left Ventricular Ejection Fraction, *SD* Standardized Difference, *STD* Standard Deviation, *WHO* World Health Organization

## Results

A total of 34,332 adult patients had preoperative echocardiographic data. The inclusion and exclusion criteria resulted in a final study population of 9420 individuals with 5.4% (*n* = 509) having reduced LVEF (Fig. 1). The study population had a mean age of 59 ± 15 years and slightly more females (52.6%) than males (47.4%) (Table 1). The mean anesthesia duration for these cases was 186.2 ± 106.0 minutes with a range of 9 to 1052 minutes. The study population was predominantly white (*n* = 7620, 80.9%), with black representing the second most common race (*n* = 921, 9.8%). Comorbidities were distributed similarly between cohorts except for coronary artery disease, arrhythmia, and congestive heart failure. Coronary artery disease, arrhythmia, and congestive heart failure were more common in patients with reduced LVEF compared to those with a normal LVEF 53% vs. 18% (SD = 0.81), 46% vs. 20% (SD = 0.61), and 67% vs. 9% (SD = 1.62).

Of the 7 medications meeting the conditions for inclusion, the use of etomidate differed most between the two cohorts (Table 2). A total of 101 (19.8%) patients with reduced LVEF received this medication while it was only used in 210 (2.4%) patients with a normal preoperative LVEF (SD = 0.58). Conversely, propofol was used less commonly in patients with reduced LVEF (80.2% vs 96.1%, SD = 0.51). Remifentanil was also used less commonly in patients with reduced LVEF (13.6% vs. 23.2%, SD = 0.25). Patients with reduced LVEF were given less fluid (747 ± 794 mL vs. 1065 ± 794 mL, SD 0.42).

**Table 2:** Comparison of intraoperative management between those with normal and reduced LVEF

	Normal LVEF	Reduced LVEF	SD
	Mean (STD)	Mean (STD)	
**Fluids**			
Fluids Balance (mL)	1065 (794)	747 (726)	0.42
	**N (%)**	**N (%)**	
**Medications**			
Calcium	695 (7.8)	69 (13.6)	0.19
Etomidate	210 (2.4)	101 (19.8)	0.58
Ephedrine	2371 (26.6)	138 (27.1)	0.01
Ketamine	848 (9.5)	28 (5.5)	0.15
Phenylephrine	4842 (54.3)	336 (66.0)	0.24
Propofol	8559 (96.1)	408 (80.2)	0.51
Remifentanil	2064 (23.2)	69 (13.6)	0.25
**Complications**			
Composite	980 (10.0)	134 (26.3)	0.25
Pulmonary	398 (4.1)	57 (11.2)	0.24
30-Day/Hospital Mortality	163 (1.7)	34 (6.7)	0.19
Acute Kidney Injury	504 (5.1)	60 (11.8)	0.24
Myocardial Injury	96 (1.0)	27 (5.3)	0.40

*LVEF* Left Ventricular Ejection Fraction, *SD* Standardized Difference, *STD* Standard Deviation

A total of 12% (*n* = 1114) of all patients experienced the primary outcome of pulmonary complications, acute kidney injury, myocardial injury, or 30-day mortality and this occurred more frequently in those with reduced LVEF (26% vs. 10%, SD = 0.25). AKI was the most common complication, occurring in 5% of patients with a normal LVEF and 12% of patients with reduced LVEF. Pulmonary complications occurred in 4% of patients with normal LVEF and 11% of patients with reduced LVEF. Mortality occurred in 1.7% (*n* = 163) with a normal LVEF and 6.7% (*n* = 34) patients with reduced LVEF. These and the other components of the composite outcome are summarized in Table 3. While the small overall number of composite outcomes in the those with moderate and severe reductions in preoperative LVEF did not allow these groups to be analyzed separately using multivariable regression, those with severely reduced LVEF did have a higher incidence of the primary outcome compared to those with moderately reduced LVEF (31.1% vs 23%, *p* < 0.0001). Similar to the Elixhauser comorbidity score, the incidence of the composite outcome was greater in those with higher ASA physical status classification scores (*p* < 0.0001). Over a third (36.9%) of ASA4 patients experienced the composite outcome while these complications only occurred in 8.7% of ASA1–3 patients. The composite outcome was more likely to occur in older patients as well, with an incidence of 10.7% in those less than 65 years and 13.9% in those 65 years or older.

**Table 3:** Characteristics of subjects with and without the primary outcome

	No Adverse Events	Composite Outcome	SD
	Mean (STD)	Mean (STD)	
**Fluids**			
Fluids Balance (mL)	1053.9 (765.1)	1004.7 (985.1)	−0.0558
Age (years)	58.5 (15.4)	61.7 (14.6)	0.2171
BMI (kg/m^2^)	29.8 (7.6)	30.1 (8.9)	0.0418
Surgery Duration (min)	113.2 (88.3)	129.1 (110.5)	0.1592
	**N (%)**	**N (%)**	
**Medication Use**			
Calcium	596 (7.2)	168 (15.1)	0.2534
Ephedrine	2278 (27.4)	231 (20.7)	−0.1569
Etomidate	229 (2.8)	82 (7.4)	0.2112
Ketamine	761 (9.2)	115 (10.3)	0.0392
Phenylephrine	4447 (53.5)	731 (65.6)	0.248
Propofol	8025 (96.6)	942 (84.6)	−0.422
Remifentanil	2015 (24.3)	118 (10.6)	−0.3663
**Subject Characteristics**			
Race			0.2656
American Indian or Alaska Native	23 (0.3)	6 (0.5)	
Asian or Pacific Islander	192 (2.3)	12 (1.1)	
Bi or Multi Racial	5 (0.1)		
Black, not of hispanic origin	804 (9.7)	117 (10.5)	
Middle Eastern	11 (0.1)	3 (0.3)	
White, not of hispanic origin	6785 (81.7)	835 (75)	
Unknown race	486 (5.9)	141 (12.7)	
Sex			0.0411
Female	4465 (53.8)	492 (44.2)	
Male	3841 (46.2)	622 (55.8)	
WHO Obesity Classification			0.1583
Underweight	148 (1.8)	38 (3.5)	
Normal Weight	2058 (25.1)	270 (25.1)	
Overweight	2613 (31.8)	335 (31.2)	
Class I Obesity	1745 (21.3)	188 (17.5)	
Class II Obesity	909 (11.1)	119 (11.1)	
Class III Obesity	740 (9)	124 (11.6)	
ASA Physical Status Classification			0.902
1	137 (1.7)	2 (0.2)	
2	2526 (30.4)	69 (6.2)	
3	4987 (60.1)	661 (59.3)	
4	652 (7.9)	382 (34.3)	
Admission Type			1.2272
Admit	3653 (44)	373 (33.5)	
Inpatient	1471 (17.7)	698 (62.7)	
Outpatient	3182 (38.3)	43 (3.9)	
Comorbidities			
CAD	1523 (18.3)	349 (31.3)	0.316
AIDS or HIV	19 (0.2)	4 (0.4)	0.0917
Alcohol Abuse	136 (1.6)	35 (3.1)	0.1305
Anemia from Blood Loss	145 (1.8)	54 (4.9)	0.1927
Arrhythmia	1520 (18.3)	490 (44)	0.5773
COPD	1479 (17.8)	380 (34.1)	0.3805
Coagulopathy	405 (4.9)	194 (17.4)	0.4107
CHF	818 (9.9)	316 (28.4)	0.4861
Iron Deficiency Anemia	384 (4.6)	123 (11)	0.2516
Depression	1165 (14)	282 (25.3)	0.2925
Diabetes	1525 (18.4)	353 (31.7)	0.3152
Fluid & Electrolyte Disorders	1142 (13.8)	570 (51.2)	0.8732
Hypertension	3925 (47.3)	781 (70.1)	0.4793
Hypothyroidism	950 (11.4)	180 (16.2)	0.1571
Liver Disease	493 (5.9)	134 (12)	0.2268
Lymphoma	272 (3.3)	51 (4.6)	0.1089
Obesity	1820 (21.9)	271 (24.3)	0.1001
Other Neurologic Disorders	540 (6.5)	194 (17.4)	0.3467
Paralysis	166 (2)	82 (7.4)	0.2673
PUD	53 (0.6)	23 (2.1)	0.1506
PVD	1041 (12.5)	234 (21)	0.2376
Psychoses	95 (1.1)	20 (1.8)	0.1027
Renal Failure	1113 (13.4)	360 (32.3)	0.4635
Solid Tumor Without Metastasis	2062 (24.8)	269 (24.2)	0.093
Surgery Risk			0.5096
Low	5268 (63.4)	449 (40.3)	
Moderate	2716 (32.7)	529 (47.5)	
High	322 (3.9)	136 (12.2)	
Duration			−0.1304
Short	5358 (64.5)	648 (58.2)	
Long	2948 (35.5)	466 (41.8)	
Surgery Type			0.5374
Acute Care & Trauma	322 (3.9)	136 (12.2)	
General Surgery	2143 (25.8)	150 (13.5)	
Neurosurgery	618 (7.4)	96 (8.6)	
Orthopedics	1156 (13.9)	105 (9.4)	
Otolaryngology	601 (7.2)	128 (11.5)	
Plastic Surgery	394 (4.7)	37 (3.3)	
Thoracic	797 (9.6)	194 (17.4)	
Urology & Gynecology	1575 (19)	157 (14.1)	
Vascular	700 (8.4)	111 (10)	

*AIDS* Acquired Immunodeficiency Syndrome, *ASA* American Society of Anesthesiologists, *BMI* Body Mass Index, *CAD* Coronary Artery Disease, *CHF* Congestive Heart Failure, *COPD* Chronic Obstructive Pulmonary Disease, *HIV* Human Immunodeficiency Virus, *PUD* Peptic Ulcer Disease, *PVD* Peripheral Vascular Disease, *SD* Standardized Difference, *STD* Standard Deviation, *WHO* World Health Organization

**Fig. 2:**
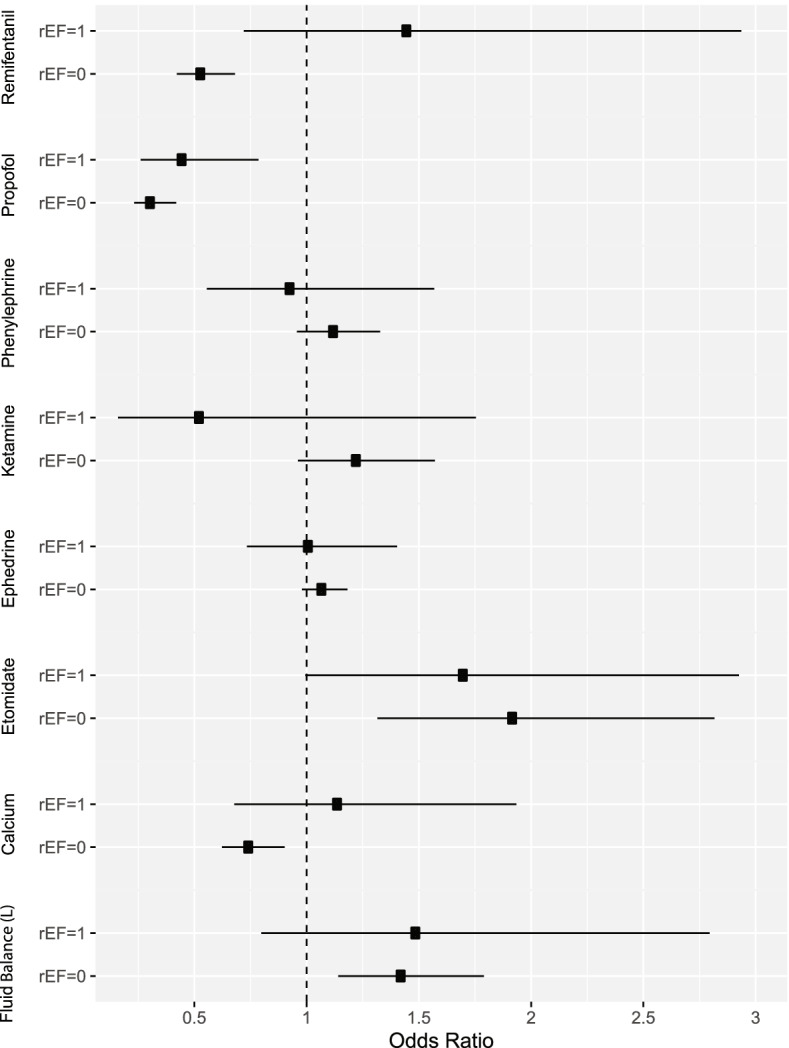
Forest plot of odds ratio for developing the primary outcome of pulmonary complications, acute kidney injury, myocardial injury, or 30-day mortality for anesthetic medications and fluid balance. This shows how subjects with reduced ejection fraction respond to intraoperative management factors compared to patients with a normal preoperative left ventricular ejection fraction (LVEF). Only remifentanil affected those with and without reduced LVEF differently. For patients with a normal preoperative LVEF, the use of remifentanil was associated with decreased odds of the primary outcome (OR 0.54, 95% CI 0.42–0.68, *p* < 0.001). However, the use of this medication in patients with reduced LVEF was associated with the opposite effect since the interaction between reduced LVEF and remifentanil use (OR 2.71, 95% CI 1.30–5.68, *p* = 0.008) means that patients with reduced LVEF have 46% greater odds of the primary outcome

Of the seven medications that were given to between 5 and 95% of studied patients, only remifentanil was associated with different outcomes between patients with reduced LVEF and normal left ventricular ejection (Fig. 2, Supplemental Table 1). After adjusting for patient and surgical characteristics, remifentanil was associated with a decreased risk of complications for the subjects with a normal LVEF (adjusted OR 0.54, 95% CI 0.42–0.68, *p* < 0.001). However, in addition to the risk associated with reduced LVEF, those who were given remifentanil intraoperatively had 2.71 times these odds compared to those with a normal LVEF (interaction term OR 2.71, 95% CI 1.30–5.68, *p* = 0.008). This translated to a 4-fold increased odds (OR 4.13, 95% CI 3.06–5.98, *p* = 0.026), of the primary outcome when patients with reduced LVEF received remifentanil as part of their anesthetic care compared to those with normal LVEF who received remifentanil.

**Fig. 3:**
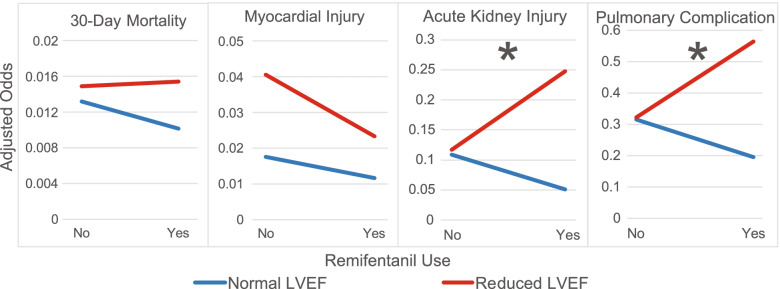
Interaction plots showing the adjusted odds of suffering 30-day mortality, myocardial injury, acute kidney injury and pulmonary complication between the use of remifentanil and presence of reduced left ventricular ejection fraction (LVEF) in patients undergoing general anesthesia for non-cardiac surgery. An asterisk denotes a significant interaction between reduced LVEF and the postoperative complication. Both acute kidney injury (OR 4.459, 95% CI 1.801–11.037, *p* = 0.0012) and pulmonary complications (OR 2.828, 95% CI 1.031–7.755, *p* = 0.0434) had a significant interaction between reduced LVEF and the use of remifentanil

Looking at the components of the composite outcome separately, remifentanil was associated with AKI and pulmonary complications, but not mortality or MI (Fig. 3, Supplemental Table 2). While the use of remifentanil was associated with a decreased incidence of pulmonary complications for patients with a normal preoperative LVEF (adjusted OR 0.62, 95% CI 0.43–0.88, *p* = 0.008), those with reduced LVEF had nearly triple the odds of this complication (interaction term OR 2.83, 95% CI 1.03–7.76, *p* = 0.043). Similarly, AKI occurred less frequently in patients with a normal LVEF who received remifentanil (adjusted OR 0.47, 95% CI 0.33–0.68, *p* < 0.001), but those with reduced LVEF had more than quadruple the risk of AKI when exposed to this medication (interaction term OR 4.46, 95% CI 1.80–11.04, *p* = 0.001).

**Fig. 4:**
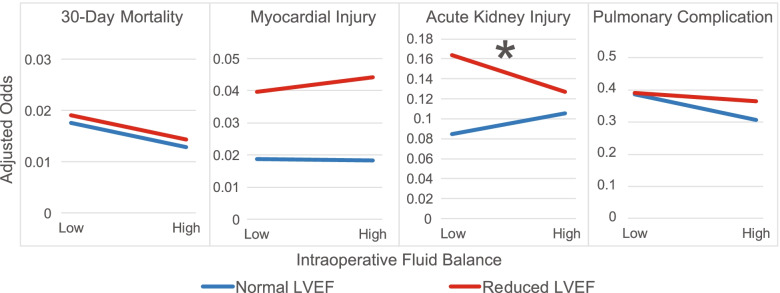
Interaction plots showing the adjusted odds of suffering 30-day mortality, myocardial injury, acute kidney injury and pulmonary complication between the intraoperative fluid balance and presence of reduce left ventricular ejection fraction (LVEF) in patients undergoing general anesthesia for non-cardiac surgery. The 25th and 75th percentiles for fluid balance are displayed. An asterisk denotes a significant interaction between reduce LVEF and the postoperative complication. This shows that, while the intraoperative fluid balance does not change the incidence of the primary outcome in individuals with a normal LVEF, reduced LVEF was associated with a higher incidence of acute kidney injury

The impact of intraoperative fluid balance on each of the individual outcomes is shown in Fig. 4. Although there was no interaction between the intraoperative fluid balance and reduced LVEF with the primary outcome, secondary outcomes were examined as an exploratory analysis. AKI was the only outcome that showed a different relationship in those with reduced LVEF compared to those with a normal LVEF. For those with a normal LVEF, a higher fluid balance was associated with 25% increased odds of AKI (OR 1.25, 95% CI 1.12–1.40, *p* < 0.001). While the relationship of fluid balance was nonlinear for those with reduced LVEF, examining the first and third quartiles of fluid balance shows that, for most fluid balances observed in the study population, reduced LVEF patients had a higher incidence of AKI when they received less fluid (interaction term OR 0.62, 95% CI 1.12–1.40, *p* = 0.035). For example, this interaction term created greater odds of AKI in those with reduced LVEF compared to those with a normal LVEF at the first fluid balance quartile (adjusted odds 0.14 vs. 0.10, respectively), while at the third quartile, the odds between these groups were nearly equal (adjusted odds 0.11 vs 0.12, respectively) (Fig. 4, Supplemental Table 3).

## Discussion

This retrospective cohort study aimed to describe if and how anesthesiologists change their care of patients with reduced LVEF and identify any treatments with a distinct risk profile in these patients. We found that etomidate, calcium, and phenylephrine were all used more in patients with reduced LVEF, while propofol, remifentanil, and fluid were used less. Only remifentanil was found to have a different effect on those with reduced LVEF compared to those with a normal preoperative LVEF. Remifentanil was associated with worse outcomes when used in those with reduced LVEF. Confounding by indication, in which an unmeasured confounder is associated with both adverse outcomes and remifentanil use, is always an issue with this study design and, while we attempted to minimize this by adjusting for multiple variables and focusing on intraoperative management features that have a significant interaction with reduced LVEF, these findings will need to be verified by future prospective studies. However, the lack of association with medications typically given to patients with a high preoperative risk of hemodynamic instability, such as etomidate, add credence to the possibility that opioid utilization may be particularly important for patients with reduced LVEF who undergo a general anesthetic as part of a non-cardiac surgical procedure.

Opioid use has important short- and long-term negative consequences such as opioid induced hyperalgesia and worsening of the opioid epidemic, respectively [16]. The use of intraoperative remifentanil has been shown to be associated with worse pain after surgery [17]. Worse pain and concomitant increased use of opioid medications is a potential mechanism underlying why remifentanil use was associated with adverse events in patients with reduced LVEF. This would be consistent with multiple studies that have shown an increased incidence of by cardiac and non-cardiac complications in patients receiving greater doses of opioids [18, 19].

The direct effect of remifentanil on cardiac function is another potential mechanism that may explain our findings. While remifentanil has minimal effect on systolic or diastolic function [20], hypotension and bradycardia, common side effects of remifentanil, may also contribute to our findings [21–23]. Patients with reduced LVEF are commonly prescribed medications such as beta-blockers and angiotensin converting enzyme inhibitors, which may increase the incidence and severity of these side effects. Patients with reduced LVEF tend to have multiple risk factors for intraoperative hypotension and bradycardia such as those described by Cheung et al.’s HEART score [24]. These would include items such as lower baseline heart rate, use of angiotensin converting enzyme inhibitors or angiotensin receptor blockers, and congestive heart failure.

The exploration of the relationship between reduced LVEF and fluid balance also revealed interesting results. While less positive fluid balance was associated with a decreased incidence of AKI in patients with a normal LVEF, those with reduced LVEF who received less fluid experienced more AKI. The association between reduced fluid and reduced AKI in the normal ejection fraction population is at odds with prospective studies comparing restrictive and liberal fluid resuscitation in patients undergoing general anesthesia [25], which may be secondary to differences in our study population and/or residual confounding. Since this confounding would likely be present in patients with reduced LVEF and normal ejection fraction, the difference we found in the way these two populations respond to resuscitation is of interest, particularly since restrictive fluid administration in patients with reduced LVEF if thought to be beneficial. Overall, this supports future studies aimed at improving our ability to guide administration of fluid to patients with reduced LVEF, particularly given the association of AKI with increased mortality, longer hospital stays, and worse patient outcomes [26–28]. While careful monitoring of salt and fluid intake, along with the prescription of diuretics, have well established roles in the care of patients with heart failure [29], different strategies may be needed during times of acute stress. It is possible that patients with reduced LVEF require more aggressive volume resuscitation during surgery to overcome pre-existing diastolic dysfunction and benefit from diuretics to speed the removal of volume given for resuscitation once the inflammatory response abates. This will need to be evaluated by prospective studies to control for the many confounding factors [30–32].

Several limitations should be considered when interpreting these results. First, like all retrospective studies, no adjustments could be made for unmeasured confounders. Therefore, additional studies that take factors not measured in this study into account are needed to confirm these findings. Second, this study only included medications that had sufficient variability in their use to detect changes between its use and adverse events. Inferences could not be made for either ubiquitously used medications such as inhaled anesthetics and opioids or rarely used medications such as epinephrine and dobutamine. Third, since these findings were obtained from a single medical center, they may not apply to institutions that have different practice patterns for how the medications included in this study are used. Therefore, more studies are needed to evaluate for associations with adverse outcomes medications in patients with reduced LVEF undergoing general anesthesia.

While these limitations and the complexity of the surgical care of patients with reduced LVEF prevent a single study from definitively answering how to best care for these patients, several attributes of this study are worth noting. By leveraging a large database of clinical information, we were able to create a large cohort of patients with reduced LVEF undergoing multiple different non-cardiac surgical procedures, which makes our finding generalizable to similar populations at other institutions. Furthermore, requiring that patients had an echocardiogram within 1 year of surgery minimized that changes that changes in their systolic function occurred prior to surgery. Finally, even though most management features were not found to have a different effect in those with and without reduced LVEF, this still supplies data that can help when deciding what medication to use when caring for these patients. For example, although propofol was used less and etomidate was used more often in patients with reduced LVEF, we did not detect a different complication rate based on the use of these medications based on the presence of reduced LVEF based on the absence of a significant interaction term.

## Conclusions

Patients with reduced LVEF are treated differently during non-cardiac surgery. The association between remifentanil and postoperative adverse events is different in those with and without reduced LVEF, where its use is associated with better outcomes in patients with normal LVEF but worse outcomes in patients with moderately or severely reduced LVEF. Future research is needed to confirm and determine if this relationship is secondary to the medication itself or if clinicians use this medication differently in this population.

## Supplementary Information

**Additional file 1: Supplemental Table 1.** Interaction of intraoperative management and reduced left ventricular ejection fraction on the occurrence of the primary outcome. **Supplemental Table 2.** Multivariable logistic regressions showing effect of remifentanil on each outcome. **Supplemental Table 3.** Multivariable logistic regressions showing effect of fluid balance on each outcome.

**Additional file 2: Supplemental Fig. 1.** Assignment of surgical risk category based on the incidence of the primary outcome for each surgical type. The surgical risk categories are displayed using distinct colors as defined in the figure legend. High-risk surgery consisted of acute care and trauma surgery. Moderate-risk surgery consisted of thoracic, vascular, otolaryngology, and neurosurgery. Low-risk surgery consisted of urology and gynecology, general surgery, orthopedics, and plastic surgery.

## Data Availability

The data that support the findings of this study are available on request from the corresponding author (M.D.M). The data are not publicly available in order to minimize the risk of loss of confidentiality of human subjects.

## Abbreviations

AKI: Acute Kidney Injury
CI: Confidence Interval
ICD: International Classifications of Disease
LASSO: Least Absolute Shrinkage and Selection Operator
LVEF: Left Ventricular Ejection Fraction
OR: Odds Ratio
RECORD: REporting of studies Conducted using Observational Routinely-collected Data
SD: Standardized Difference
STD: Standard deviation
STROBE: Strengthening the Reporting of Observational Studies in Epidemiology
VIF: Variance Inflation Factor
