# Supplementary material for: Identification of intraoperative management strategies that have a differential effect on patients with reduced left ventricular ejection fraction: a retrospective cohort study
Source: BMC Anesthesiol. 2022 Sep 10;22:288. doi: 10.1186/s12871-022-01817-z (PMC9463783; doi:10.1186/s12871-022-01817-z)
Supplement: Supplementary file 1 — Additional file 1: Supplemental Table 1. Interaction of intraoperative management and reduced left ventricular ejection fraction on the occurrence of the primary outcome. Supplemental Table 2. Multivariable logistic regressions showing effect of remifentanil on each outcome. Supplemental Table 3. Multivariable logistic regressions showing effect of fluid balance on each outcome. [file 12871_2022_1817_MOESM1_ESM.docx]

# Supplemental Tables

**Supplemental Table 1.** Interaction of intraoperative management and reduced left ventricular ejection fraction on the occurrence of the primary outcome

| **Calcium** | **OR** | **95% CI** | **P-Value** |
| --- | --- | --- | --- |
| Reduced LVEF | 1.30 | (0.98, 1.71) | 0.065 |
| Calcium | 1.43 | (1.14, 1.79) | 0.002 |
| Reduced LVEF *Calcium | 1.05 | (0.54, 2.02) | 0.895 |
| **Ephedrine** |  |  |  |
| Reduced LVEF | 1.19 | (0.89, 1.60) | 0.248 |
| Ephedrine | 0.75 | (0.62, 0.90) | 0.002 |
| Reduced LVEF *Ephedrine | 1.53 | (0.88, 2.66) | 0.134 |
| **Etomidate** |  |  |  |
| Reduced LVEF | 1.19 | (0.89, 1.59) | 0.241 |
| Etomidate | 1.92 | (1.32, 2.82) | 0.001 |
| Reduced LVEF *Etomidate | 0.89 | (0.46, 1.71) | 0.719 |
| **Ketamine** |  |  |  |
| Reduced LVEF | 1.39 | (1.07, 1.80) | 0.012 |
| Ketamine | 1.23 | (0.96, 1.57) | 0.099 |
| Reduced LVEF *Ketamine | 0.43 | (0.13, 1.46) | 0.177 |
| **Phenylephrine** |  |  |  |
| Reduced LVEF | 1.50 | (0.95, 2.37) | 0.081 |
| Phenylephrine | 1.13 | (0.96, 1.33) | 0.151 |
| Reduced LVEF *Phenylephrine | 0.83 | (0.48, 1.42) | 0.496 |
| **Propofol** |  |  |  |
| Reduced LVEF | 0.83 | (0.47, 1.44) | 0.504 |
| Propofol | 0.31 | (0.23, 0.42) | <0.001 |
| Reduced LVEF *Propofol | 1.45 | (0.78, 2.71) | 0.240 |
| **Remifentanil** |  |  |  |
| Reduced LVEF | 1.15 | (0.88, 1.51) | 0.300 |
| Remifentanil | 0.54 | (0.42, 0.68) | <0.001 |
| Reduced LVEF *Remifentanil | 2.71 | (1.30, 5.68) | 0.008 |

Each model adjusted for age, BMI, sex, postoperative hemoglobin, surgical risk, surgery duration, and Elixhauser comorbidity score. A standardized difference >0.1 was used to identify variables for inclusion in multivariable models.

CI = Confidence Interval, LVEF = Left Ventricular Ejection Fraction OR = Odds Ratio

# Supplemental Table 2

**Supplemental Table 2.** Multivariable logistic regressions showing effect of remifentanil on each outcome.

| **Composite Outcome** | **OR** | **95% CI** | **P-Value** |
| --- | --- | --- | --- |
| Reduced LVEF | 1.15 | (0.88, 1.51) | 0.300 |
| Remifentanil | 0.54 | (0.42, 0.68) | <0.001 |
| Reduced LVEF *Remifentanil | 2.71 | (1.30, 5.68) | 0.008 |
| Age (years) | 1.00 | (1.00, 1.01) | 0.092 |
| BMI (kg/m^2^) | 1.04 | (1.03, 1.04) | <0.001 |
| Female Sex | 0.91 | (0.84, 0.98) | 0.009 |
| Postoperative Hemoglobin (g/dL) | 0.82 | (0.79, 0.85) | <0.001 |
| High Surgical Risk | 1.45 | (1.23, 1.71) | <0.001 |
| Moderate Surgical Risk | 1.10 | (0.98, 1.24) | 0.098 |
| Long Duration | 0.99 | (0.92, 1.06) | 0.702 |
| Elixhauser Comorbidity Score | 1.06 | (1.05, 1.06) | <0.001 |
| **30- Day Mortality** |  |  |  |
| Reduced LVEF | 1.13 | (0.68, 1.86) | 0.640 |
| Remifentanil | 0.77 | (0.47, 1.26) | 0.298 |
| Reduced LVEF *Remifentanil | 1.35 | (0.30, 6.05) | 0.691 |
| Age (years) | 1.02 | (1.01, 1.04) | <0.001 |
| BMI (kg/m^2^) | 0.99 | (0.97, 1.02) | 0.622 |
| Female Sex | 0.98 | (0.83, 1.15) | 0.803 |
| Postoperative Hemoglobin (g/dL) | 0.85 | (0.78, 0.93) | <0.001 |
| High Surgical Risk | 1.21 | (0.86, 1.70) | 0.266 |
| Moderate Surgical Risk | 1.60 | (1.26, 2.03) | <0.001 |
| Long Duration | 0.71 | (0.59, 0.84) | <0.001 |
| Elixhauser Comorbidity Score | 1.07 | (1.06, 1.09) | <0.001 |
| **Myocardial Injury** |  |  |  |
| Reduced LVEF | 2.31 | (1.37, 3.88) | 0.002 |
| Remifentanil | 0.66 | (0.35, 1.24) | 0.198 |
| Reduced LVEF *Remifentanil | 0.87 | (0.140, 5.31) | 0.881 |
| Age (years) | 1.02 | (1.00, 1.03) | 0.032 |
| BMI (kg/m^2^) | 1.00 | (0.97, 1.03) | 0.820 |
| Female Sex | 0.95 | (0.79, 1.16) | 0.625 |
| Postoperative Hemoglobin (g/dL) | 0.88 | (0.80, 0.98) | 0.014 |
| High Surgical Risk | 1.32 | (0.89, 1.95) | 0.172 |
| Moderate Surgical Risk | 1.21 | (0.91, 1.60) | 0.191 |
| Long Duration | 0.98 | (0.81, 1.19) | 0.856 |
| Elixhauser Comorbidity Score | 1.04 | (1.02, 1.05) | <0.001 |
| **Acute Kidney Injury** |  |  |  |
| Reduced LVEF | 1.082 | (0.76, 1.54) | 0.661 |
| Remifentanil | 0.47 | (0.33, 0.68) | <0.001 |
| Reduced LVEF *Remifentanil | 4.46 | (1.80, 11.04) | 0.001 |
| Age (years) | 1.00 | (0.99, 1.00) | 0.348 |
| BMI (kg/m^2^) | 1.03 | (1.02, 1.04) | <0.001 |
| Female Sex | 0.87 | (0.79, 0.96) | 0.006 |
| Postoperative Hemoglobin (g/dL) | 0.9 | (0.86, 0.95) | <0.001 |
| High Surgical Risk | 1.02 | (0.81, 1.27) | 0.885 |
| Moderate Surgical Risk | 0.84 | (0.72, 0.99) | 0.034 |
| Long Duration | 1.24 | (1.12, 1.36) | <0.001 |
| Elixhauser Comorbidity Score | 1.05 | (1.04, 1.06) | <0.001 |
| **Pulmonary Complications** |  |  |  |
| Reduced LVEF | 1.02 | (0.70, 1.50) | 0.923 |
| Remifentanil | 0.62 | (0.43, 0.88) | 0.008 |
| Reduced LVEF *Remifentanil | 2.83 | (1.03, 7.76) | 0.043 |
| Age (years) | 1.00 | (0.99, 1.01) | 0.552 |
| BMI (kg/m^2^) | 1.04 | (1.03, 1.05) | <0.001 |
| Female Sex | 0.95 | (0.85, 1.06) | 0.361 |
| Postoperative Hemoglobin (g/dL) | 0.76 | (0.71, 0.80) | <0.001 |
| High Surgical Risk | 2.05 | (1.68, 2.49) | <0.001 |
| Moderate Surgical Risk | 1.33 | (1.14, 1.55) | <0.001 |
| Long Duration | 0.76 | (0.67, 0.85) | <0.001 |
| Elixhauser Comorbidity Score | 1.05 | (1.04, 1.06) | <0.001 |

BMI=Body Mass Index, CI=Confidence Interval, LVEF = Left Ventricular Ejection Fraction, OR=Odds Ratio. A standardized difference >0.1 was used to identify variables for inclusion in multivariable models.

# Supplemental Table 3

**Supplemental Table 3.** Multivariable logistic regressions showing effect of fluid balance on each outcome.

| **Composite Outcome** | **OR** | **95% CI** | **P-Value** |
| --- | --- | --- | --- |
| Reduced LVEF | 1.39 | (0.99, 1.97) | 0.061 |
| Fluid Balance | 1.08 | (0.98, 1.18) | 0.126 |
| Reduced LVEF *Fluid Balance | 0.94 | (0.68, 1.31) | 0.733 |
| Age (years) | 1.01 | (1.00, 1.01) | 0.072 |
| BMI (kg/m^2^) | 1.04 | (1.03, 1.04) | <0.001 |
| Female Sex | 0.90 | (0.84, 0.97) | 0.006 |
| Postoperative Hemoglobin (g/dL) | 0.81 | (0.78, 0.84) | <0.001 |
| High Surgical Risk | 1.52 | (1.29, 1.79) | <0.001 |
| Moderate Surgical Risk | 1.04 | (0.93, 1.17) | 0.458 |
| Long Duration | 0.95 | (0.87, 1.03) | 0.215 |
| Elixhauser Comorbidity Score | 1.06 | (1.05, 1.06) | <0.001 |
| **30- Day Mortality** |  |  |  |
| Reduced LVEF | 1.09 | (0.58, 2.03) | 0.792 |
| Fluid Balance | 0.73 | (0.55, 0.96) | 0.025 |
| Reduced LVEF *Fluid Balance | 1.02 | (0.45, 2.29) | 0.964 |
| Age (years) | 1.02 | (1.01, 1.04) | <0.001 |
| BMI (kg/m^2^) | 0.99 | (0.97, 1.02) | 0.581 |
| Female Sex | 0.97 | (0.82, 1.14) | 0.679 |
| Postoperative Hemoglobin (g/dL) | 0.86 | (0.79, 0.94) | 0.001 |
| High Surgical Risk | 1.22 | (0.87, 1.71) | 0.253 |
| Moderate Surgical Risk | 1.52 | (1.20, 1.92) | 0.001 |
| Long Duration | 0.78 | (0.64, 0.94) | 0.011 |
| Elixhauser Comorbidity Score | 1.07 | (1.06, 1.08) | <0.001 |
| **Myocardial Injury** |  |  |  |
| Reduced LVEF | 2.11 | (1.06, 4.17) | 0.033 |
| Fluid Balance | 0.97 | (0.74, 1.26) | 0.806 |
| Reduced LVEF *Fluid Balance | 1.15 | (0.61, 2.18) | 0.667 |
| Age (years) | 1.01 | (1.00, 1.03) | 0.036 |
| BMI (kg/m^2^) | 1.00 | (0.98, 1.03) | 0.769 |
| Female Sex | 0.94 | (0.78, 1.14) | 0.546 |
| Postoperative Hemoglobin (g/dL) | 0.87 | (0.79, 0.97) | 0.009 |
| High Surgical Risk | 1.34 | (0.91, 1.99) | 0.141 |
| Moderate Surgical Risk | 1.15 | (0.87, 1.52) | 0.322 |
| Long Duration | 0.98 | (0.79, 1.22) | 0.877 |
| Elixhauser Comorbidity Score | 1.04 | (1.02, 1.05) | <0.001 |
| **Acute Kidney Injury** |  |  |  |
| Reduced LVEF | 1.94 | (1.25, 3.02) | 0.003 |
| Fluid Balance | 1.25 | (1.12, 1.40) | <0.001 |
| Reduced LVEF *Fluid Balance | 0.62 | (0.40, 0.97) | 0.035 |
| Age (years) | 1.00 | (0.99, 1.00) | 0.439 |
| BMI (kg/m^2^) | 1.03 | (1.02, 1.04) | <0.001 |
| Female Sex | 0.88 | (0.79, 0.96) | 0.007 |
| Postoperative Hemoglobin (g/dL) | 0.89 | (0.85, 0.94) | <0.001 |
| High Surgical Risk | 1.08 | (0.86, 1.34) | 0.525 |
| Moderate Surgical Risk | 0.80 | (0.69, 0.94) | 0.006 |
| Long Duration | 1.13 | (1.01, 1.26) | 0.029 |
| Elixhauser Comorbidity Score | 1.05 | (1.04, 1.06) | <0.001 |
| **Pulmonary Complications** |  |  |  |
| Reduced LVEF | 1.01 | (0.63, 1.63) | 0.952 |
| Fluid Balance | 0.79 | (0.67, 0.94) | 0.007 |
| Reduced LVEF *Fluid Balance | 1.17 | (0.69, 1.00) | 0.555 |
| Age (years) | 1.00 | (0.99, 1.01) | 0.539 |
| BMI (kg/m^2^) | 1.04 | (1.03, 1.05) | <0.001 |
| Female Sex | 0.94 | (0.84, 1.05) | 0.245 |
| Postoperative Hemoglobin (g/dL) | 0.76 | (0.71, 0.80) | <0.001 |
| High Surgical Risk | 2.10 | (1.73, 2.56) | <0.001 |
| Moderate Surgical Risk | 1.25 | (1.08, 1.46) | 0.004 |
| Long Duration | 0.80 | (0.71, 0.91) | 0.001 |
| Elixhauser Comorbidity Score | 1.04 | (1.03, 1.05) | <0.001 |

BMI = body mass index, CI=Confidence Interval, LVEF = Left Ventricular Ejection Fraction, OR=Odds Ratio. A standardized difference >0.1 was used to identify variables for inclusion in multivariable models.
