# Supplementary material for: Identification of intraoperative management strategies that have a differential effect on patients with reduced left ventricular ejection fraction: a retrospective cohort study
Source: BMC Anesthesiol. 2022 Sep 10;22:288. doi: 10.1186/s12871-022-01817-z (PMC9463783; doi:10.1186/s12871-022-01817-z)
Supplement: Supplementary file 2 — Additional file 2: Supplemental Fig. 1. Assignment of surgical risk category based on the incidence of the primary outcome for each surgical type. The surgical risk categories are displayed using distinct colors as defined in the figure legend. High-risk surgery consisted of acute care and trauma surgery. Moderate-risk surgery consisted of thoracic, vascular, otolaryngology, and neurosurgery. Low-risk surgery consisted of urology and gynecology, general surgery, orthopedics, and plastic surgery. [file 12871_2022_1817_MOESM2_ESM.pdf]

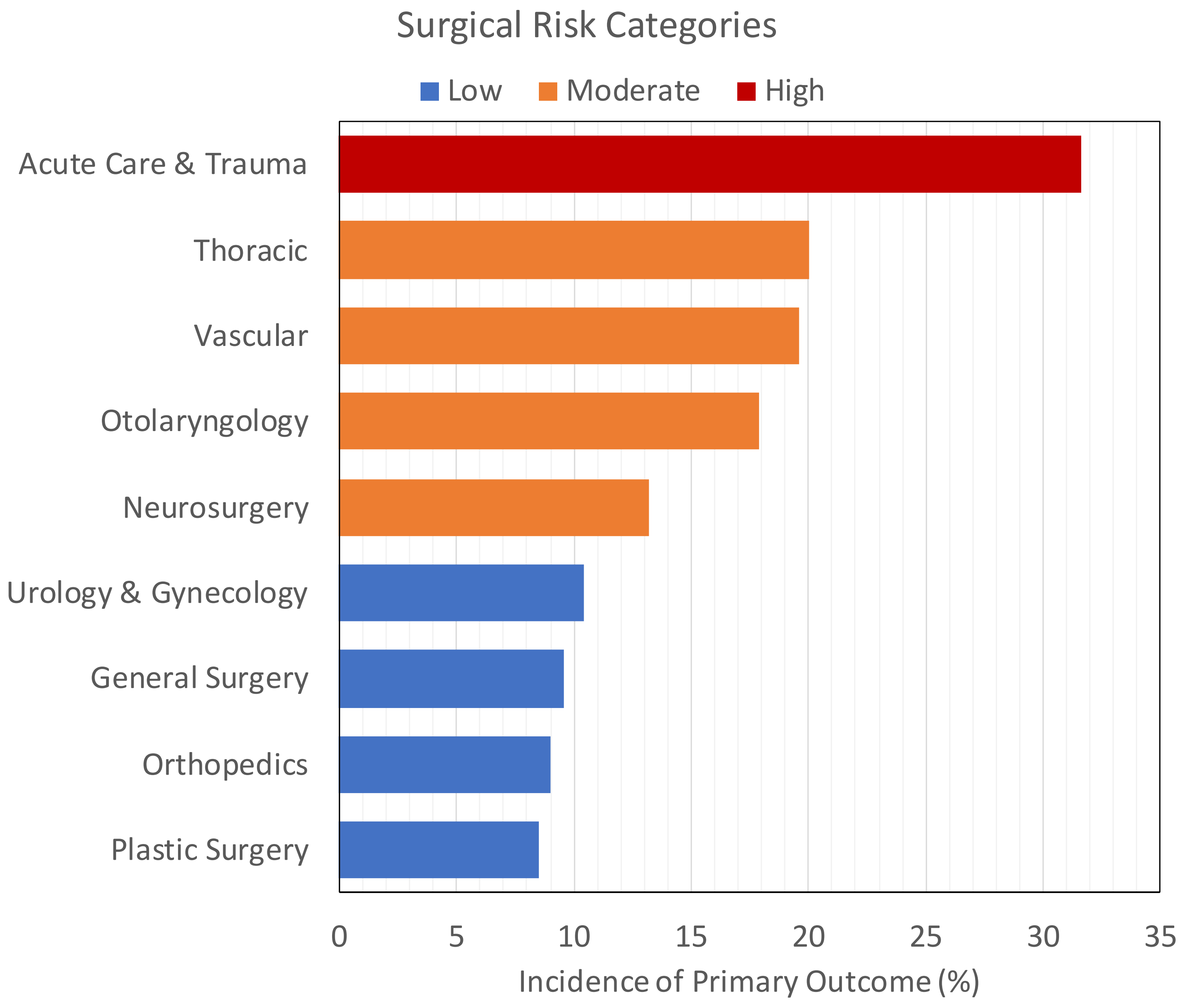

Supplemental Figure 1. Assignment of surgical risk category based on the incidence of the primary outcome for each surgical type. The surgical risk categories are displayed using distinct colors as defined in the figure legend. High-risk surgery consisted of acute care and trauma surgery. Moderate-risk surgery consisted of thoracic, vascular, otolaryngology, and neurosurgery. Low-risk surgery consisted of urology and gynecology, general surgery, orthopedics, and plastic surgery.
